# Spontaneous Coronary Artery Dissection and Its Management: A Case Report

**DOI:** 10.7759/cureus.25474

**Published:** 2022-05-30

**Authors:** Arsh N Patel, Jaydip Desai, Parth K Patel, Fady Wanna, Jason Cox

**Affiliations:** 1 Department of Research, Alabama College of Osteopathic Medicine, Dothan, USA; 2 Department of Research, George Washington University School of Medicine and Health Sciences, Washington, DC, USA; 3 Department of Cardiothoracic Surgery, Flowers Medical Group, Dothan, USA

**Keywords:** coronary artery bypass grafting (cabg), left main coronary artery, intra-aortic balloon pump, percutaneous coronary intervention, spontaneous coronary artery dissection

## Abstract

We illustrate a notable case of an elderly male presenting to a community hospital with six out of 10 substernal chest discomfort and electrocardiogram changes consistent with an anterolateral myocardial infarction. Percutaneous Coronary Intervention (PCI) was initiated following aspirin and anticoagulation administration, which further revealed a critical distal left main spontaneous coronary artery dissection (SCAD). Cardiothoracic and Vascular Surgery consults led to the recommendation of emergent two-vessel Coronary Artery Bypass Grafting (CABG). The patient’s clinical status resolved to full recovery and was discharged on postoperative day five. The incidence of SCAD in older men has not been well documented in current literature. Prevalence in older males is 0.02%. However, it rises to 10.8% in females less than 50 years of age and with acute coronary syndromes (ACS) and ST-segment elevation. Our aim is to incorporate this case report into the current literature and help improve early diagnosis and treatment based on current recommendations.

## Introduction

Spontaneous coronary artery dissection (SCAD) is a rare condition characterized by a non-iatrogenic, non-traumatic separation of the coronary vessel wall layers. A false lumen gets created between either the tunica intima and media or the tunica media and externa [1,2]. Blood will continue to flow despite the coronary artery tear; however, if hemorrhage into the opening occurs, it may stagnate and thrombose. There, it adds pressure onto the true lumen wall and forces a dissection. The obstruction caused by the compressed lumen can reduce blood flow and lead to myocardial ischemia and subsequent infarction. The first recorded case of SCAD was documented early in 1931 [3]. Since its onset, recent literature has helped refine clinical presentation and management. The degree of occlusion typically correlates to the severity of the symptoms associated with SCAD. These symptoms can range from asymptomatic presentation to unstable angina, acute myocardial infarction, and possibly ventricular arrhythmias leading to sudden cardiac death [1].

SCAD currently has a prevalence rate of 0.2% in a population of 11,605 patients from an interventional cardiology laboratory database. Of those with SCAD, about 0.07% is found in men and 0.6% in women [3]. Remarkably, younger women with cardiac histories or Fibromuscular Dysplasia (FMD) are significantly more likely to experience SCAD. Female patients less than 50 years old with ACS or with ACS and ST-segment elevation have a prevalence rate of 8.7% and 10.8%, respectively [3]. This SCAD case is notable as the afflicted patient is both male and elderly-aged, two traits correlated with low prevalence levels. The patient presented in our case with typical angina symptoms that lead to PCI, eventually leading to a diagnosis of SCAD. After emergent two-vessel CABG was performed, he progressed well through short- and now long-term post-operative care. Our aim is to highlight a spontaneous and rare case of SCAD with atypical risk factors using a prior literature review.

## Case presentation

A 61-year-old Caucasian male was transferred to the emergency department via ambulance with a chief complaint of “aching” six out of ten substernal chest discomfort. His past medical history is significant for controlled hypertension, hyperlipidemia, osteoarthritis, and gastroesophageal reflux disease (GERD). His pertinent home medications included valsartan-hydrochlorothiazide and hydrocodone-ibuprofen. Previous surgical history included a spinal stimulator placement for chronic back pain and a Nissen fundoplication for GERD. He is a former cigarette smoker but stated his last tobacco product use was in 1986. He infrequently consumes alcohol and denies any previous illicit substance use. He lives a physically active lifestyle due to his highly demanding job in landscaping despite having a BMI of 28 kg/m^2^.

**Figure 1:**
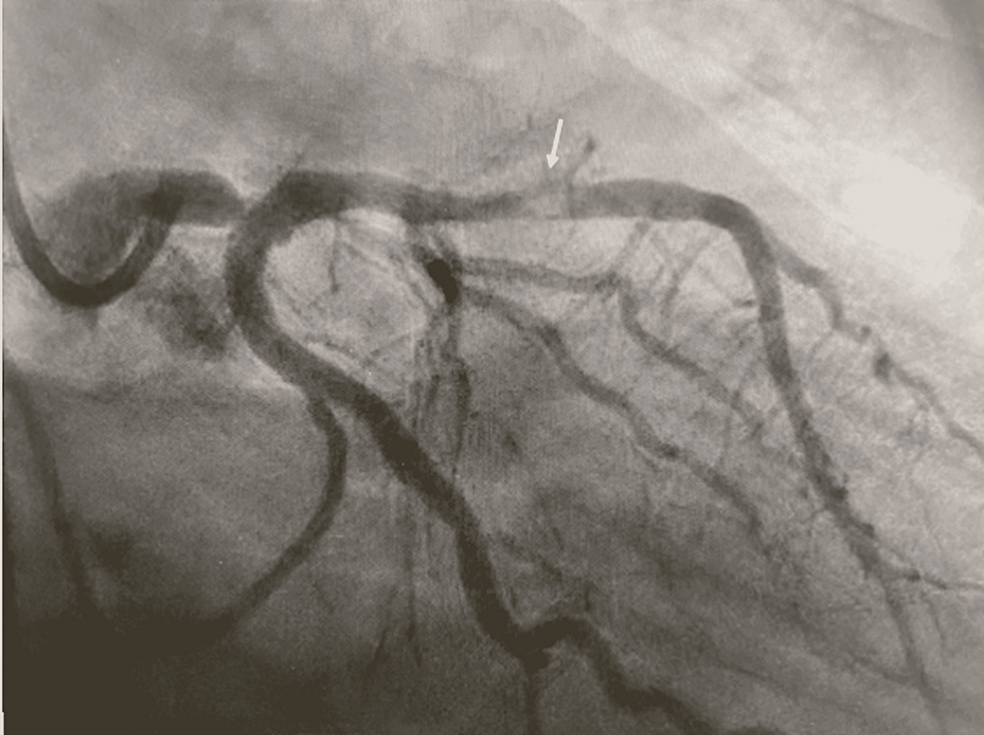
Distal Left Main SCAD The white arrow is pointing to the Spontaneous Coronary Artery Dissection diagnosed during the catheterization which required emergent two-vessel coronary artery bypass grafting (CABG). Noted is a critical proximal left anterior descending (LAD) artery stenosis.

Upon initial presentation, the electrocardiogram (EKG) revealed ST-segment elevations in leads I, aVL, V2, V3, and V4 along with reciprocal J-point depressions in leads II, III, and aVF. The EKG changes were consistent with an anterolateral myocardial infarction and warranted further emergent intervention via cardiac catheterization. The patient was immediately started on aspirin and heparin therapy in the emergency room and was then transferred to the catheterization lab for emergent investigation. With the modified Seldinger technique, right femoral access was obtained as the guidewire and catheter proceeded proximally. An ectatic aortic root was noted on ascension to the coronary vessels, and the Q catheter (Maquet Getinge Group, Rastatt, Germany) engaged the left coronary artery (LCA) while the JR4 catheter (Maquet Getinge Group, Rastatt, Germany) engaged the right coronary artery (RCA). Diagnostic studies revealed a two-vessel disease with a critical distal left main spontaneous coronary artery dissection occluding approximately 90% of the vessel and a critical proximal left anterior descending (LAD) artery stenosis (Figure 1 and Video 1). Incidentally, the diagnostic study revealed a Kugel artery which is a rare anatomical variant of the coronary artery vasculature with an anastomotic connection between the RCA and left circumflex artery [4]. Cardiothoracic and Vascular Surgery was consulted for emergent two-vessel coronary artery bypass grafting (CABG). Pre-operatively to the CABG, an intra-aortic balloon pump (IABP; Maquet Getinge Group, Rastatt, Germany) was placed through a right femoral approach by the Interventionalist in the proximal descending aorta.

**Video 1:** Video of Cardiac Catheterization showing Left Main SCAD Here we capture a real-time video using contrast-dye to show the Left Main SCAD at 25% speed of the original footage. SCAD: Spontaneous Coronary Artery Dissection

Prior to cardiothoracic surgery, the Society of Thoracic Surgeons' (STS) Risk Calculator from the STS Adult Cardiac Surgery Database was computed, and the results were discussed with the patient. Risk scores showed a 3.606% risk of operative mortality, 21.057% risk of prolonged ventilation, 3.040% risk of re-operation, and an overall morbidity/mortality risk of 28.804%. After the isoelectric arrest was accomplished, a two-vessel CABG was successfully anastomosed using the Left Internal Mammary Artery (LIMA) to LAD and a saphenous vein graft to the mid-Obtuse Marginal (MOM) artery. After anastomoses, the patient was given protamine sulfate for reversal of the heparin drip and return of preoperative hemodynamic status was achieved with no complications. The pericardium was reconstructed, and the sternum was closed with the usual Figure-of-8 wires. The patient tolerated the procedure well without any complications and was discharged to the intensive care unit.

Post-operatively, the patient had an uneventful recovery with only minimal lower extremity edema and peri-operative anemia. The plan of action was to slowly begin weaning the patient off the IABP. By postoperative day two, the IABP frequency was 50% augmentation with stable hemodynamics and eventually was discontinued later that same day. The patient was started on in-patient aspirin, clopidogrel, and enoxaparin. In-patient physical therapy was consulted on postoperative day three for evaluation and treatment in order to guide the patient to return to the prior level of functioning safely. By postoperative day four, the patient was ambulating close to 100% without any assisted device or difficulty while maintaining appropriate sternal precautions. He achieved complete stability by postoperative day five and was prepared for discharge with education regarding lifestyle modification for risk factors. He was additionally begun on amiodarone, aspirin, atorvastatin, metoprolol tartrate, and hydrocodone-acetaminophen prior to discharge. Future plans for outpatient follow-up will include discussions regarding the addition of angiotensin-converting enzyme (ACE) inhibitors or angiotensin II receptor blockers (ARBs) to improve long-term patient outcomes.

## Discussion

**Figure 2:**
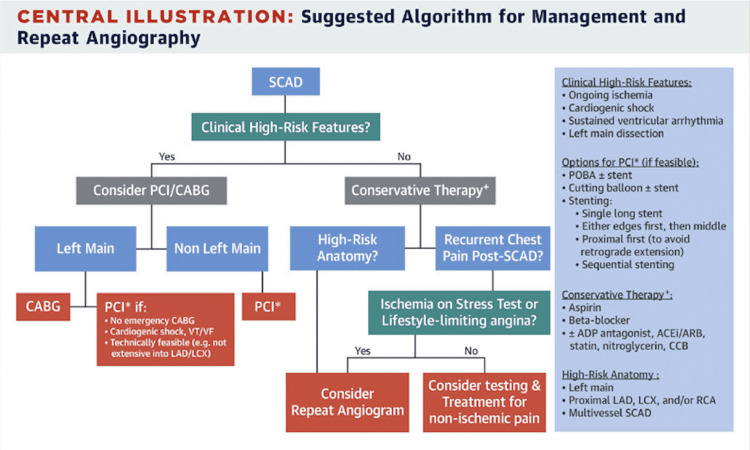
Suggested Treatment Algorithm for SCAD Work-up Source: [5] "Reprinted from JACC: Cardiovascular Interventions, Vol 12 /Issue 6, Saber Hassan, Roshan Prakash, Andrew Starovoytov, Jacqueline Saw, Natural History of Spontaneous Coronary Artery Dissection With Spontaneous Angiographic Healing, Pages 518-527, Copyright (2022), with permission from Elsevier."

The low incidence and prevalence of SCAD events make recognition and medical management two of the most important aspects of achieving positive patient outcomes. The case presented outlines typical features of the acute coronary syndrome (ACS) accompanied by atypical patient demographics and the ultimate outcome followed by a suggested treatment algorithm (Figure 2) [5].

**Figure 3:**
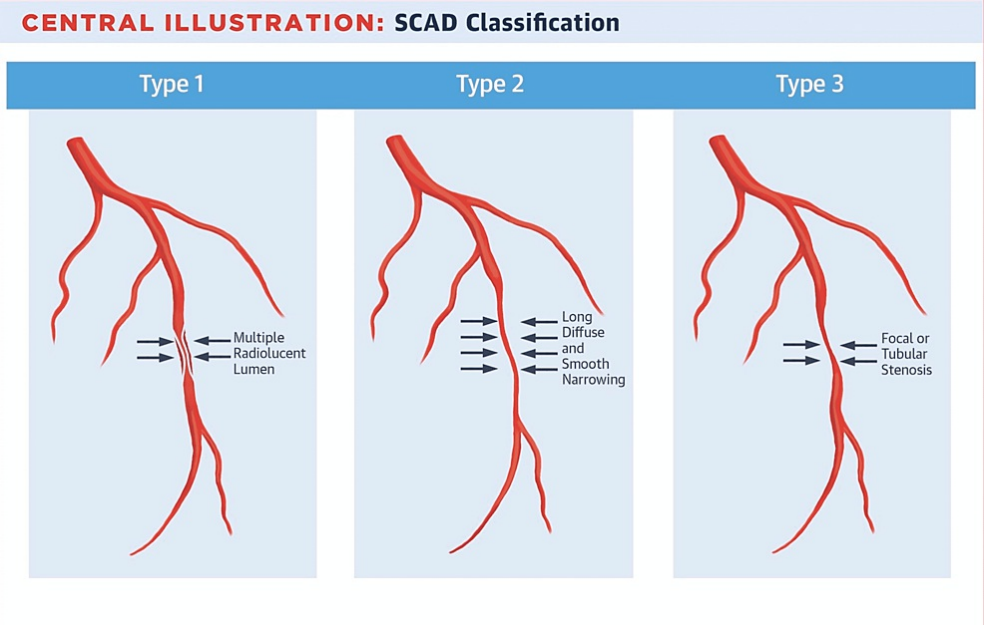
Classification system of different types of SCAD Source: [7] "Reprinted from JACC, Vol 70 /Issue 9, Jacqueline Saw, Karin Humphries, Eve Aymong, Tara Sedlak, Roshan Prakash, Andrew Starovoytov, G.B. John Mancini, Spontaneous Coronary Artery Dissection Clinical Outcomes and Risk of Recurrence, Pages 1148-1158, Copyright (2022), with permission from Elsevier."

Since symptoms of SCAD may be indistinguishable from other causes of ACS, a physician should have increased levels of suspicion when patients present with sudden onset of symptoms during isometric exercise or under emotional distress; however, it still remains a challenge to make a clear-cut diagnosis [6]. Current literature supports invasive coronary angiography for initial diagnosis, but there may be uncertainties that remain. For those cases with uncertain diagnostic exploration, intracoronary imaging and cardiac magnetic resonance imaging have been found useful [6]. Classification of SCAD can be subdivided into three main types as outlined by Saw et al. (Figure 3) [7].

As mentioned above, the demographic impacted by SCAD is “typically” women of reproductive age with symptoms of ACS related to predisposing factors such as fibromuscular dysplasia (FMD), pregnancy, multiparity, connective tissue disease, and chronic inflammatory conditions which create increased wall tension in the coronary artery leading to progressive tunica intima or media degeneration [3,8]. Out of the possible risk factors mentioned, FMD has shown the strongest association with SCAD [9]. This association further supports the predominance of SCAD in women of reproductive age as FMD is a non-inflammatory, non-atherosclerotic disorder of medium-sized arterial beds seen mostly in that respective demographic. In their most recent case series, Yip and Saw analyzed 168 patients impacted by SCAD with approximately 72% of the patients impacted by FMD, which correlates to a previous retrospective study conducted by Mayo Clinic that found a 50% association [9]. Interestingly, a post-mortem and ante-mortem case study of 83 patients outlined by DeMaio et al. showed predominantly dissection of the left coronary artery system in 85% of the patients [10]. 

An alternative diagnosis to consider in ACS presentations similar to SCAD may be Takotsubo Cardiomyopathy (TTC). TTC is a condition that is believed to occur due to a sudden catecholamine surge from physical or emotional stress, more often seen in females. Despite the intense physical demands of our patient’s occupation, the cardiac angiogram showed no evidence of left ventricular apical ballooning in the absence of significant coronary stenosis. Buccheri and Zambelli explored a link between SCAD and TTC via analysis of published case reports, ultimately hypothesizing a possible causative relationship between SCAD leading to TTC or vice versa [11]. However, there needs to be further explorative studies and meta-analyses examining a true correlation. Nonetheless, one should keep an open mind when creating a differential diagnosis for SCAD as TTC should be a possible consideration in appropriate circumstances.

There are several treatment modalities for SCAD such as conservative measurements, percutaneous coronary intervention (PCI), and CABG. A Howard University Hospital meta-analysis of 440 patients with single lesion SCAD in the LCA or RCA systems from 1931 to 2008 showed that patients had better outcomes with early aggressive therapy using stent placement or CABG compared to conservative therapy (p=0.006, p=0.023, respectively) [12]. However, treatment for SCAD is dependent on the location of arterial dissection and occlusion. Regragui et al. argue in their case report that conservative therapy should be first-line therapy for hemodynamically and clinically stable patients regardless of which coronary artery vessel is occluded [13]. Additionally, Alfonso et al. explained that watchful waiting is more of an appropriate decision due to the adverse risks PCI may cause, including the extension of the coronary dissection, guidewire passage into the false lumen, or hematoma occlusion [14]. The long-term prognosis for patients with SCAD has not been well defined despite the various interventions that could be performed. In-hospital and long-term outcomes from an observational multicenter study of 134 documented SCAD patients demonstrated no significant findings in conservative versus revascularization management [15]. In the retrospective cohort study conducted comparing SCAD versus non-SCAD cohorts by Clare et al., a total of 26,598 ACS patients were analyzed and 208 patients were diagnosed with SCAD [16]. The study evaluated both short-term and one-year prognosis of patients with SCAD versus non-SCAD ACS. Patients treated for SCAD via coronary catheterization showed a better thirty-day mortality rate of 1.4% compared to 4.1% in the non-SCAD cohort, as well as one-year outcomes showing 2.4% versus 8.8% mortality rate favoring the SCAD group (p<0.001) [16]. Additional observational studies should be considered to get a stronger foundation regarding the long-term prognosis of SCAD, as well as further insights into optimal treatment modalities. 

Patients that develop SCAD are expected to manifest a wide variety of symptoms during multiple follow-ups in their lives. Cardiovascular-related events that occur include chest pain, anxiety, depression, recurrent myocardial infarctions, congestive heart failure, stroke, and recurrent SCAD. Major adverse cardiac events were reported in 15 to 37% between a five-to-seven-year interval [17]. The recurrence rate of SCAD is debated in the literature. In subsequent de novo dissections, SCAD recurs in previously unaffected arteries about 77 to 100% of the time [17]. In a trial performed at the Mayo Clinic, SCAD recurred in about 15 of 87 patients 2.8 years after the initial onset [17]. Both short- and long-term prognoses following SCAD can be highly worrisome for both the patient and physician provider. Therefore, it is imperative that a multi-disciplinary approach between vascular surgery, cardiothoracic surgery, cardiology, and internal medicine be used when managing encounters with suspected SCAD.

## Conclusions

We present an interesting case of an elderly-aged male with minimal risk factors developing SCAD in the left main coronary artery requiring aggressive therapy with CABG. On presentation at the emergency room, the patient experienced typical ACS symptoms along with ST-segment elevations correlating to an anterolateral myocardial infarction, and upon further investigation using angiography SCAD was diagnosed. SCAD is often an underdiagnosed subcategory of ACS which requires immediate recognition and initiation of therapy to improve long-term patient success. Limited case reports due to low disease incidence allow this case to boost current literature in order to improve early recognition and outcomes of SCAD among physicians with high levels of clinical suspicion.
